# High rate of non-vaccine targeted high-risk HPV genotypes circulate among women in Eastern Ethiopia

**DOI:** 10.1038/s41598-024-51594-7

**Published:** 2024-01-10

**Authors:** Ayichew Seyoum, Berhanu Seyoum, Tadesse Gure, Ashenafi Alemu, Dawit Hailu Alemayehu, Addisu Alemu, Anteneh Belachew, Dessalegn Abeje Tefera, Abraham Aseffa, Rawleigh Howe, Andargachew Mulu, Adane Mihret

**Affiliations:** 1 College of Health and Medical Sciences, Haramaya University, Harar, Ethiopia; 2 Armauer Hansen Research Institute, Addis Ababa, Ethiopia

**Keywords:** Epidemiology, Molecular biology

## Abstract

The World Health Organization [WHO] recommends a genotype-specific human papillomavirus [HPV] vaccination as a primary prevention strategy to control the burden of cervical cancer globally. In Ethiopia, where the non-vaccine-targeted HPV genotypes have not been adequately studied, a vaccination initiative was launched in 2018 targeting HPV-6,-11, -16, and -18 for girls aged 14–18 years. The co-existence of both vaccine-targeted and non-targeted genotypes is a serious concern, as it can accelerate cancer progression. Therefore, this study was conducted to determine the prevalence of non-vaccine-targeted HPV genotypes and assess the level of multiple infections with other genotypes in eastern Ethiopia. A health facility-based cross-sectional study including 110 women with positive HPV DNA results was conducted from April to August 2021. A structured questionnaire to collect demographic and clinical data was used. Cervical swabs were collected using L-shaped FLOQSwabs. Women's cytological profile was determined based on Pap smear test results. An automated nucleic acid extraction system using STARMag 96 ProPrep Universal Extraction Kit was utilized following the manufacturer's protocol. An amplification assay in real-time was employed to amplify and identify the HPV Late 1 [L1] gene, which is utilized for genotyping purposes. Following this, the collected data was entered into Epi data version 3.1 software, and the analysis was performed using STATA version 14. A total of 110 women [age range 30–60 years, mean age = 36.5 years and SD ± 6.9] had positive HPV DNA results and were included in the study. Among these, 108 women had valid co-testing [Pap test and HPV DNA test] results for further analysis, and the results of the remaining 2 women were rejected. Overall, the prevalence of non-vaccine-targeted HPV was 56 (51.8%, 95%CI [0.42, 0.61]), of which 28 women (25.4%, 95%CI [0.18, 0.34]) had a single non-vaccine HPV genotype infection. The remaining 29 women (26.4%, 95% CI: 0.190–0.355) experienced multiple infections. The non-vaccine-targeted genotypes of HPV-35 accounted for 11 cases (10%, 95%CI [0.06, 0.17]), HPV-68 was detected in 9 women (8.2%, 95%CI [0.04, 0.15]), HPV-56 and HPV-66 were both found in 8 cases each (7.3%, 95%CI [0.04, 0.14]) of the total. In addition, out of these 108 women, 93 (86.1%, 95%CI [0.78, 0.91]) had low-grade squamous intraepithelial lesions, 13 (12%, 95%CI [0.07, 0.20]) no intraepithelial lesion or malignancy, and two (1.9%, 95%CI [0.01, 0.07]) high-grade squamous intraepithelial lesions. Furthermore, there was no statistical difference [*p* = 0.755] between vaccine-targeted and non-vaccine-targeted genotypes as the primary cause of cervical lesions. In conclusion, the findings of the present study highlight the existence of a notable prevalence of multiple infections caused by non-vaccine-targeted HPV genotypes. Therefore, it is recommended that both the Federal and regional health bureaus to evaluate the range of hr HPV genotypes protected by the current HPV vaccine and explore the option of transitioning from the quadrivalent HPV vaccine to a novavalent vaccine that includes seven high-risk HPV genotypes.

## Introduction

Human papillomavirus [HPV] is one of the most common carcinogens that cause cervical cancer^1^. Despite most infections clearing up within 2 years without showing any symptoms, HPV genital infection can lead to different types of clinical diseases^2^. The number of HPV genotypes listed by the International Agency for Research on Cancer [IARC] is continuously increasing and has reached 229 [www.hpvcenter.se, on 12/11/2023]^3,4^.

Approximately 40 genotypes of HPV have the ability to infect the reproductive organs of both males and females, including the genital skin, vulva [the external area surrounding the vagina], anus, vaginal lining, cervix, and uterus^5^. The IARC has recently classified 12 HPV genotypes as carcinogenic, including HPV-16, HPV-18, HPV-31, HPV-33, HPV-35, HPV-39, HPV-45, HPV-51, HPV-52, HPV-56, HPV-58, and HPV-59 [group 1]. HPV-68 is also considered probably carcinogenic or group 2A. Additionally, HPV-26, HPV-30, HPV-34, HPV-53, HPV-66, HPV-67, HPV-69, HPV-70, HPV-73, HPV-82, HPV-85, and HPV-97 have been linked to rare causes of cervical cancer and are classified as possibly carcinogenic [group 2B]^6–8^. Globally, HPV-16 and HPV-18 are responsible for approximately 70% of cervical cancer cases.

Persistent infection caused by high-risk HPV [hr HPV] is present in nearly all cases of cervical cancer^6^. However, the level of persistence, progression to high-grade squamous intraepithelial lesion [HSIL], and cervical cancer depends on the hr HPV genotype^9^. Globally, cervical cancer is the fourth most common cause of cancer in women and its prevalence of the disease is very high among low- and middle-income countries [LMIC]^10–12^. Countries are using two preventive measures to prevent the spread of the virus. The first level of prevention is the HPV vaccine and the second level of prevention is the cervical cancer screening program. Although these are effective prevention methods, their application only helps to prevent cervical cancer^13,14^.

The *papillomaviridae* displays a well-preserved genome structure, with its 8 genes being expressed in a controlled manner within squamous epithelia, both spatially and temporally. These genes include six early genes [E1, E2, E4, E5, E6, and E7] and two late genes [L1 and L2]. Additionally, the long control region [LCR] acts as the origin of replication and regulates transcriptional activities^15^. The expression of L1 through recombinant methods in various systems results in the formation of virus-like particles [VLPs] that lack genetic material. Animal challenge studies have demonstrated that immunization with L1 VLPs offers strong protection, although limited to specific types. At present, there are six approved vaccines against Human Papillomavirus, all containing L1-only VLPs derived from the most clinically significant genotypes^16^.

Although growing HPV in tissue culture is challenging, its genotyping can be determined by analysing the nucleotide sequence on the L1 gene^17,18^. The molecular assays of HPV are capable of partial, extended, or complete genotyping of the virus^19–21^. Partial genotyping is the ability of an assay to report HPV-16 and HPV-18 individually and the remaining 12 h HPV genotypes in one group while extended genotyping refers to assays that individually detect at least 6 h HPV genotypes and the remaining in one or more groups. In addition, complete genotyping reflects assays that report all hr HPV genotypes individually^19^.

All the vaccines that have been used so far are very effective in preventing infections caused by HPV-16 and HPV-18. In addition to these two genotypes, quadrivalent vaccines are highly effective in preventing two additional warts-causing HPV genotypes, HPV-6 and HPV-11. In addition to HPV-16 and HPV-18, the nonavalent vaccine is highly effective against the two low-risk [HPV-6 and HPV-1] and five hr HPV genotypes [HPV-31, HPV-33, HPV-45, HPV-52, and HPV-58]^16,22^. While the vaccine offers protection against a significant percentage [70–80%] of HPV infections, there is still a potential threat of HPV-related diseases from the remaining strains, even after receiving the vaccination^23,24^. The non-vaccine-targeted HPV genotypes, HPV-35/ HPV-39/ HPV-51/ HPV-56/ HPV-59, account for 7% of cervical cancers worldwide^25^. However, in some areas, it was found that their prevalence in women aged 24–45 years is from 5.1% in the Philippines^26^ to 48.6%^27^.

In 2018, Ethiopia launched a program to vaccinate girls aged 14–18 against HPV-6, -11, -16, and -18. However, there has been limited research on other HPV genotypes not targeted by the vaccine in the country^28^. The results of previous vaccine trials and post-implementation evaluations showed that the bivalent^29^ and quadrivalent vaccines provided some additional levels of protection against additional HPV genotypes not directly targeted by the vaccines. But, the level of cross-protection varies with vaccine type, HPV genotype, and study^30^. While some countries have seen a decrease in HPV prevalence among women after implementing vaccination programs, there has been no significant change in the prevalence of non-vaccine-targeted HPV^31,32^. In addition to the low-level and inconsistent cross-protection, cervical cancer is often associated with multiple HPV genotype infections. Furthermore, cervical cancer is often associated with multiple HPV genotype infections, and non-vaccine-targeted genotypes may lead to the development of cancer more rapidly and extensively when infected alongside vaccine-targeted genotypes [For example HPV-51 and HPV-58%]^33–35^.

In order to implement an effective and timely strategy for preventing cervical cancer in Ethiopia, it is crucial to address the scarcity of research-based data regarding the prevalence of non-vaccine-targeted HPV and the extent of multi-infection. Therefore, to tackle the lack of information and contribute valuable data for future research on non-vaccine-targeted HPV, the current study was conducted to determine the prevalence of non-vaccine-targeted HPV, the occurrence level of multiple infections, and associated factors.

## Materials and methods

### Study setting and design

The study was undertaken in two administrative towns [Harar and Jigjiga] situated in two regions and one administrative council [Dire-Dawa], which were included in the nine regions and two administrative councils of Ethiopia during the research period. It was conducted from April to August 2021 at three hospitals: Hiwot Fana Comprehensive Specialized University Hospital in Harar, Dil-Chora Referral Hospital in Dire-Dawa, and Shiek Hassan Yabare Referral Hospital in Jigjiga, located in Eastern Ethiopia. The selection of these hospitals was based on the presence of cervical cancer screening services and the availability of specialized professionals in gynecology and pathology for clinical diagnosis and cytology examinations.

### Population and eligibility criteria

This study included women who met the following inclusion criteria and who sought medical care for gynecological problems. Women who have lived permanently in the study area for at least 6 months, aged between 30 and 65 years^14,36^ and signed their consent to participate in the study were included in the study. However, women who have had sexual intercourse within the 24 h prior to clinical examination, or who have had heavy menstrual bleeding and it is difficult to perform an appropriate screening test, or who have had physical or mental problems for various reasons and who have had difficulty conducting interviews, or who have had a history of gynecological surgery were not included in this study.

### Sample size

The sample size for initial cervical cancer screening using visual inspection with acetic acid [VIA] was calculated using OpenEpi303 software. The assumptions to calculate the sample size for cross-sectional study of a double population proportion were: 95% confidence interval, 80% power, ratio of unexposed [Normal cytology]/exposed [abnormal cytology] is 2, percent of unexposed with outcome is 71%^37^, percent of exposed with outcome is 80.4%^38^ and odds ratio is 1.7. Taking into account a 15% non-response rate, the final sample size was determined to be 901, which was initially calculated as 784.

Out of the 901 individuals who participated in the study and underwent DNA tests, all 110 women who tested positive were included in the study and conveniently underwent genotyping.

### Operational definitions

Cervarix vaccine-targeted high-risk HPV genotypes are the hr HPVs genotypes which are targeted and able to be prevented by the Cervarix vaccine. These are HPV-16 and HPV-18.

High-risk HPV genotypes: HPV genotypes, such as HPV types 16, 18, 31, 33, 35, 39, 45, 51, 52, 56, 58, 59, 66, and 68, possess high-risk characteristics due to their oncogenic potentials. These high-risk HPV genotypes are responsible for the emergence of cervical cancer and its precursor lesions.

Nonavalent vaccine-targeted high-risk HPV genotypes are the hr HPVs genotypes which are targeted and able to be prevented by the Nonavalent vaccine. These are HPV-6, HPV HPV-11, HPV-16, HPV-18, HPV-31, HPV-33, HPV-45, HPV-52, and HPV-58).

Non-vaccine targeted high-risk types are the hr HPVs not targeted and prevented directly by Cervarix or Gardasil or Gardasil 9 vaccines. These are HPV-35, HPV-39, HPV-51, HPV-56, HPV-59, and HPV-68.

Quadrivalent vaccine-targeted high-risk HPV genotypes are the hr HPVs genotypes which are targeted and able to be prevented by the Quadrivalent vaccine. These are HPV-6, HPV-11, HPV-16 and, HPV-18.

Unsatisfactory Pap test result means a clinical specimen that does not have enough cells or a specimen in which the cells appear clumped together or are obscured by blood or mucus and are difficult to examine.

### Data collection

Trained midwives collected socio-demographic and other relevant bio-behavioral and clinical data through a face-to-face interview. This interview utilized a carefully designed and pre-tested structured questionnaire, which was created using information from reputable peer-reviewed journals. To validate the questionnaire, a pilot study was conducted at Jugol hospital. Gynecologists performed visual inspections of the cervix and collected liquid-based cervical swabs, while pathologists conducted cytological examinations under microscopy. In addition, pathology, microbiology, and other relevant data that could not be directly obtained from the study participants were collected through a checklist based on hospital records books.

### Cytological examinations

Women who initially sought medical assistance for gynecologic problems resembling HPV infection and fulfilled the inclusion criteria underwent screening based on the WHO's guidelines for visual inspection with acetic acid [VIA]^39^. The exfoliated ectocervical and endocervical cells were collected using L-shaped Endo/Esocervical FLOQSwab [Copan Italia SpA, Brescia, Italy] and used to make a smear on the slide. The smear was fixed on the slide using ethanol and stained according to standard protocols^40^. Then, the cytological features of cells were read and results were recorded on standardized forms according to the 2015 Bethesda System^41^. Additional descriptions related to cytological test result reporting and decision-making can be seen in our research work published earlier^42^

### Liquid-based cervical swabs collection and storage

The L-shaped FLOQSwabs [Copan Italia SpA, Brescia Italy] were used to collect cervical swabs from the cervical canal, and these swabs were then placed into a 2 mL tube of eNAT medium following the instructions provided by the manufacturer. The medium can store the collected nucleic acids for a month at room or higher temperature for HPV DNA detection and genotyping. The collected cervical samples were transported to Child Health and Mortality Surveillance [CHAMPS] Ethiopia project, Haramaya University, and Armauer Hansen Research Institute [AHRI], Addis Ababa laboratories and stored at -80^O^C until further processed.

### HPV DNA extraction, detection and genotyping

The molecular laboratory work was conducted at AHRI laboratories in Addis Ababa. A 200 μL aliquot of cervical swab was subjected to nucleic acid extraction using the STARMag 96 ProPrep Kit from Seegene, Korea. The extraction was performed on the SEEPREP32 automated Liquid Handling Workstation, which is a bead transfer-based nucleic acid extraction instrument for in vitro diagnostics. This workstation can extract up to 32 specimens in just 30 min. Finally, the extracted DNA was eluted with 70 μL of elution buffer.

HPV detection and genotyping were carried out following the instructions provided by the manufacturer, utilizing the Anyplex II HPV HR kit [Seegene, Seoul, Korea] and a CFX96 Deep Well real-time thermocycler [Bio-Rad, Hercules, CA, USA].

A novel technology known as Oligonucleotide Cleavage and Extension [TOCE], originating from Seoul, Korea, has emerged for the detection of multiple targets in the same fluorescence channel on real-time PCR instruments. This technology involves the utilization of two components, namely ‘Pitcher’ and ‘Catcher’^43,44^. The target-specific primer is extended by Taq polymerase and encounters the target-bound Pitcher, which is subsequently cleaved by 5’ nuclease activity, resulting in the release of the tagging region. The released label portion exhibits complementarity to the portion held by the catcher.

The Catcher duplex in the TOCE assay is designed with a specific pre-defined target sequence, allowing for the evaluation of its melting temperature and enabling the detection of 14 HPV genotypes through multiplex real-time PCR. To ensure the efficiency of DNA purification, PCR inhibition, and cell adequacy, the L1 gene of HPV and human beta-globin are co-amplified as internal controls. The detection and genotyping of the 14 HPV types are carried out in five fluorescent channels [FAM, HEX, Cal Red 610, Quasar 670, and Quasar 705], with individual parameters set for each channel to ensure target detection and validity.

### Quality control measures

The questionnaire was initially developed based on previously published research articles in peer-reviewed journals and was written in English. It was then translated into Afan Oromo, Amharic, and Somali by local language experts to facilitate communication with study participants, conducted a pilot study, and reviewed for scientific validity. Data collectors received comprehensive training, and two pathologists examined slides interchangeably.

Midwives were extensively trained on data quality, study objectives, and proper data format and storage. Two pathologists carefully analyzed stained slides to determine women's cytological condition. Both experts alternately examined samples and collaboratively resolved discrepancies for consistency and quality.

A pilot study at Jugol Hospital confirmed the effectiveness of the eNAT UTM in preserving, transporting, and collecting nucleic acids. Cervical swabs were obtained from 45 women, and seven random samples were selected. Nucleic acid was extracted using the Qiagen EZ1 Advanced XL workstation, and the presence of the ERV-3 gene was detected in all seven samples.

### Statistical methods

Before entering the data into the database, measures were taken to ensure that it was complete. Then, the data was cleaned and coded before being inputted into Epi data version 3.1 software [The EpiData Association in Odense, Denmark]. Subsequently, the data was exported to STATA version 14 [Stata Corp LP. in College Station, Texas, USA] for further analysis.

The study population and relevant variables were described using frequencies, proportions, and summary statistics. Proportions were calculated for categorical and discrete variables, and the significance level of values was determined using the Pearson Chi-squared and Fisher's exact test when required. In order to determine the factors associated with prevalence of non-vaccine targeted hr HPV infection, the binary logistic regression model was utilized. The level of association was assessed using a *p*-value and a 95% confidence interval. Variables with *p* < 0.25 were selected as potential candidates for multivariate logistic regression, while variables with *p* < 0.05 were considered to have statistically significant associations.

### Ethical considerations

The study protocol got ethical approval from the Institutional Health Research Ethics Review Committee [IHRERC] of the College of Health and Medical Sciences, Haramaya University, Ethiopia [Reference Number: IHRERC/212/2020] and the Armauer Hansen Research Institute Ethics Committee [Reference Number: PO/20/20]. Before data collection, we obtained signed informed consent from the study participants. During the course of the study, we linked women with positive HPV DNA in their samples to an Oncologist and Gynecologist for optimal and ongoing treatment. We also coded the names and other identifiers of study participants to protect the confidentiality of the results. The study was conducted in accordance with the Helsinki Declaration^45^ and national research ethics review guidelines of Ethiopia^46^.

## Results

### Socio-demographic characteristics and disease composition of women

Out of the 901 women who underwent Pap test and DNA testing to screen for HPV, it was determined that 110 [12.2%] women tested positive for HPV. All 110 samples underwent genotyping, and the majority of the individuals participating in the study, 99 [90%] women were residents of urban areas, 81 were married [73.6%], 70 were aged 18 years or older [63.5%], and 89 were reported having multiple sexual partners [80.9%]. Additionally, 80 [72.7%] women had a single HPV genotype [Table 1].

**Table 1:** An overview of the sociodemographic and disease profile of women residing in Ethiopia in 2021.

Variable	Category	Single infection, N [%]	Multiple infections, N [%]	Total, N [%]
Residence	Urban	71 [88.7]	28 [93.3]	99 [90]
	Rural	9 [11.2]	2 [6.7]	11 [10]
Age [in years]	< 30	25 [31.2]	8 [26.7]	33 [30]
	31—35	22 [27.5]	8 [26.7]	30 [27.3]
	36—40	21 [26.2]	4 [13.3]	25 [22.7]
	41—45	5 [6.2]	6 [20]	11 [10]
	46—50	4 [5]	3 [10]	7 [6.4]
	56—60	3 [3.7]	1 [3.3]	4 [3.6]
Marital status	Married	60 [75]	21 [70]	81 [73.6]
	Never married	6 [7.5]	3 [10]	9 [8.2]
	Widowed	3 [3.7]	3 [10]	6 [4.4]
	Divorced	10 [12.5]	3[10]	13 [11.8]
	Separated	1 [1.2]	–	1 [0.9]
Age at first Marriage [ in years]	< 15	7 [8.7]	4 [13.3]	11 [10]
	15—17	19 [23.7]	10 [33.3]	29 [26.5]
	> 18	54 [67.5]	16 [3.3]	70 [63.5]
Current occupational status	Full time employee	22 [27.5]	9 [30]	31 [28.2]
	Part-time employee	9 [11.2]	4 [13.3]	13 [11.8]
	Unemployed	42 [52.5]	13 [43.3]	55 [50]
	Student	2 [2.5]	2 [6.7]	4 [3.6]
	Retired	1 [1.2]	1 [3.3]	2 [1.8]
	Other	4 [5]	1 [3.3]	5 [4.5]
Educational status	Unable to read and write	35 [43.7]	8 [26.7]	43 [39.1]
	Between 1 to 8 grades	23 [28.7]	13 [43.3]	36 [32.7]
	Between 9 to 12 grades	10 [12.5]	4 [13.3]	14 [12.7]
	Diploma and above	12 [15]	5 [16.7]	17 [15.4]
Number of sexual partners	Single	17 [21.2]	4 [13.3]	21 [19.1]
	Multiple	63 [78.7]	26 [86.7]	89 [80.9]

A single infection means that only one type of HPV is detected at the time of testing, and multiple infection means that more than one type of HPV is present at the time of testing.

### The distribution of hr HPV genotype proportions among women

The study used DNA testing to detect and type HPV from 110 cervical swabs. The top five genotypes were HPV-16 (32.7%, 95%CI [0.24, 0.42]), HPV-31 (19.1%, 95%CI [0.13, 0.28]), HPV-52 (11.8%, 95%CI [0.07, 0.19]), HPV-58 (10.9%, 95%CI [0.06, 0.18]), and HPV-35 (10%, 95%CI [0.06, 0.17]). Among these, HPV-35 is not targeted by the vaccine.

**Figure 1:**
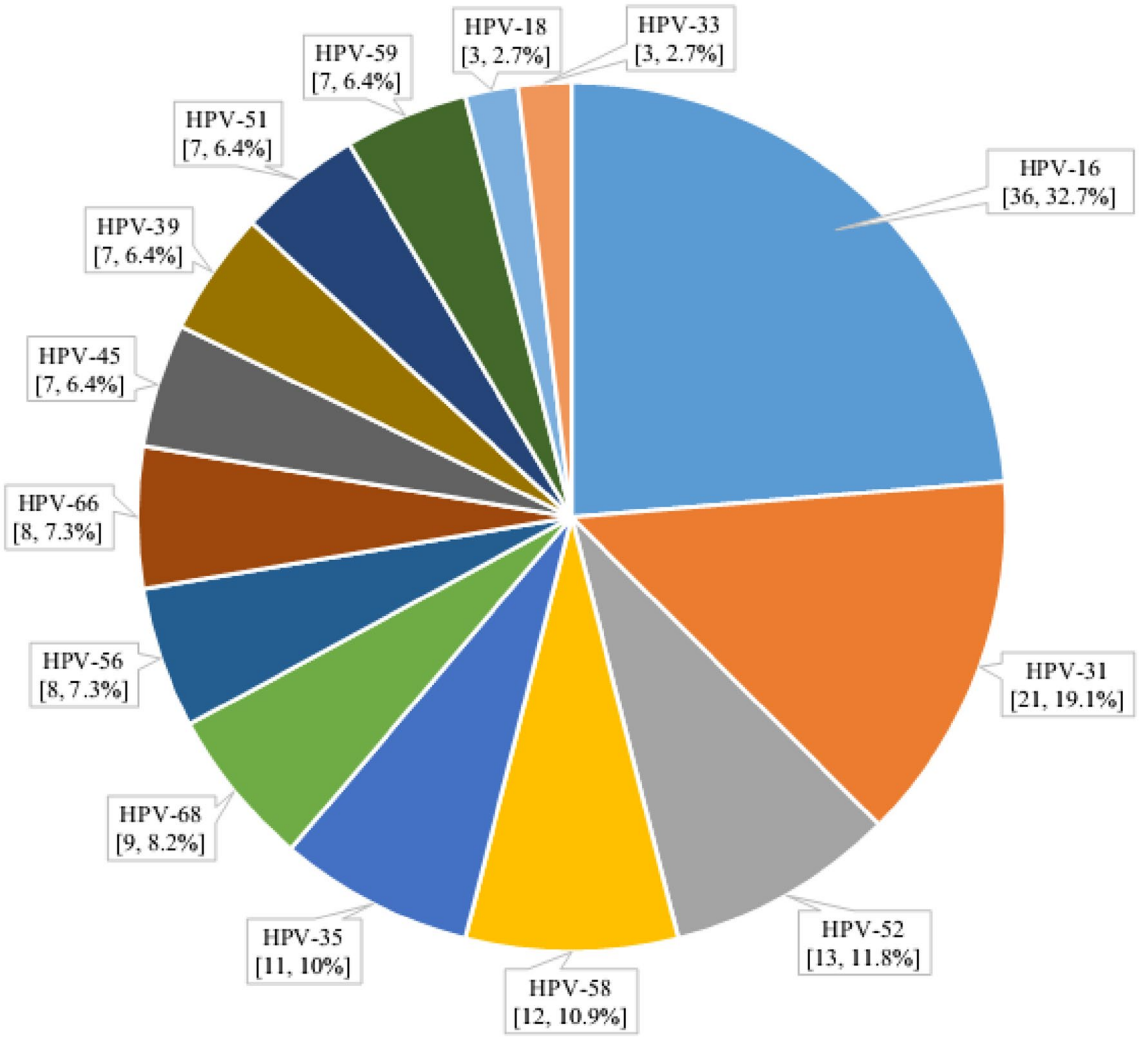
The distribution of vaccine and non-vaccine targeted hr HPV genotype proportions among the study participants.

Following HPV-35, the next three highest-ranking non-vaccine-targeted genotypes were HPV-68 (8.2%, 95%CI [0.04, 0.15]), HPV-56 (7.3%, 95%CI [0.04, 0.14]), and HPV-66 (7.3%, 95%CI [0.04, 0.14]) (Fig. 1).

### Non-vaccine-targeted genotypes, types of infection, and cytological profiles

Besides the HPV genotypes that can be prevented through vaccines approved by WHO namely Cervarix, Gardasil, and Gardasil 9, we detected other non-vaccine-targeted HPV genotypes [HPV-35, HPV-39, HPV-51, HPV-56, HPV-66, and HPV-68] from women who participated in the study.

Thus, among the 57 women (51.8%, 95%CI [0.42, 0.61]) with non-vaccine-targeted genotypes [including multiple infection], 28/110 (25.4%, 95%CI [0.18, 0.34]) women had a single non-vaccine-targeted hr HPV genotype infection, while the remaining 29/110 (26.4%, 95%CI [0.19, 0.35]) women had multiple infection. In addition, HPV-35 (10%, 95%CI [0.06, 0.17]), HPV-68 (8.2%, 95%CI [0.04, 0.15]), HPV-56 (7.3%, 95%CI [0.04, 0.14]), and HPV-66 (7.3%, 95%CI [0.04, 0.14]) were found in higher numbers (Table 2).

**Table 2:** Non-vaccine-targeted genotypes distribution based on types of infections.

hr HPV* [n = 110]			95%CI	Single infection			Multiple infection		
Genotype	N	%		N	%	95%CI	N	%	95%CI
HPV-35	11	10	[0.05, 0.17]	3	2.7	[0.01, 0.08]	8	7.3	[0.04, 0.14]
HPV-39	7	6.4	[0.03, 0.13]	4	3.6	[0.01, 0.09]	3	2.7	[0.01, 0.08]
HPV-51	7	6.4	[0.03, 0.13]	5	4.5	[0.02, 0.11]	2	1.8	[0.01, 0.07]
HPV-56	8	7.3	[0.04, 0.14]	3	2.7	[0.01, 0.08]	5	4.5	[0.02, 0.11]
HPV-59	7	6.4	[0.04, 0.14]	4	3.6	[0.01, 0.09]	3	2.7	[0.01, 0.08]
HPV-66	8	7.3	[0.04, 0.14]	4	3.6	[0.01, 0.09]	4	3.6	[0.01, 0.09]
HPV-68	9	8.2	[0.04, 0.15]	5	4.5	[0.02, 0.11]	4	3.6	[0.01, 0.09]

*high-risk Human papillomavirus.

Among 110 women who participated in the study to determine the prevalence of vaccine and non-vaccine-targeted genotypes based on cytology results, two women were excluded from further analyses due to unsatisfactory Pap test results. Therefore, we used the test results of 108 women for further analysis.

**Figure 2:**
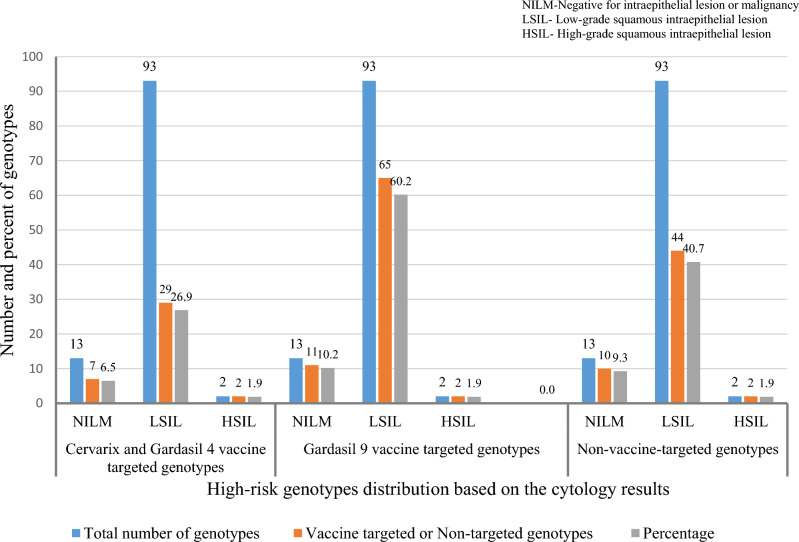
Vaccine and non-vaccine targeted genotypes distribution based on the cytology results.

Among the 108 women, 93 (86.1%, 95%CI [0.78, 0.91]) women had Low-grade squamous intraepithelial lesions [LSIL], 13 (12%, 95%CI [0.07, 0.19]) women had no intraepithelial lesion or malignancy [NILM], and two (1.9%, 95%CI [0.00, 0.07]) women had cytological results of high-grade squamous intraepithelial lesions [HSIL] (Fig. 2).

A total of 157 h HPV genotypes [including multiple infections] were detected in 110 women. Of the women with hr HPV detected, 30 (27.3%, 95%CI [0.20, 0.36]) women had multiple infections while the remaining were analysed for single infection.

Among women with multiple infections, the occurrence of vaccine-targeted hr HPV-16 [16.7%] and hr HPV-31 [6.7%] with the non-vaccine targeted genotypes and the non-vaccine targeted hr HPV-35 [6.7%] with other vaccine-targeted genotype, placed them in the top three positions (Table 3).

**Table 3:** The occurrence of HPV-16, HPV-31, and HPV-33 with other genotypes among the study participants.

Genotype	Other genotypes detected with HPV-16			Number [n = 30]
HPV-16*	HPV-31*	HPV-52*	HPV-66**	1
HPV-16*	HPV-31*	HPV-68**		1
HPV-16*	HPV-45*	HPV-59**		1
HPV-16*	HPV-51**	HPV-59**		1
HPV-16*	HPV-52*	HPV-56**		1
HPV-16*	HPV-45*			2
HPV-16*	HPV-31*			1
HPV-16*	HPV-58*			1
HPV-31*	HPV-58*			3
HPV-31*	HPV-33*			2
HPV-31*	HPV-66**			2
HPV-31*	HPV-39**			1
HPV-31*	HPV-45*			1
HPV-35**	HPV-39**	HPV-52*	HPV-58*	1
HPV-35**	HPV-56**	HPV-66**		1
HPV-35**	HPV-52*			1
HPV-35**	HPV-56**			1
HPV-35**	HPV-68**			2

*Vaccine-targeted hr HPV genotype.

**Non-vaccine-targeted hr HPV genotype.

### Non-vaccine targeted genotypes association with cervical lesions

In addition to descriptive statistics, we examined the association between the incidence of cervical lesions and the vaccine- and non-vaccine-targeted genotypes. Accordingly, we found no statistically significant difference between vaccine-targeted and non-vaccine-targeted genotypes that could be classified as a primary cause of cervical lesions [*p* = 0.755]. This means that non-vaccine-targeted genotypes are equally likely to be classified as primary causes of cervical lesions as vaccine-targeted genotypes. Thus, it is important to consider the prevalence of non-targeted genotypes in vaccines.

### Vaccine-targeted genotypes based on types of infections

In addition to the non-vaccine-targeted hr HPV genotypes, the study has also determined the three vaccines [Cervarix, Gardasil, and Gardasil 9] targeted hr HPV genotypes.

Accordingly, HPV-16 and -18 were detected in a total of 39 (35.4%, 95%CI [0.27, 0.45]) women. Among these, HPV-16 was detected in 36 (32.7%, 95%CI [0.24, 0.42]) while HPV-18 was detected in only 3 (2.7%, 95%CI [0.01, 0.08]) women. Additionally, 25 [22.7%] of 36 women with HPV-16 had a single infection (95%CI [0.16, 0.32]) and in the remaining 11 [10%] women it was found to be multiple infections (95%CI [0.05, 0.17]).

Similarly, the hr HPV genotypes targeted by nonavalent vaccine, namely HPV-16, HPV-18, HPV-31, HPV-33, HPV-45, HPV-52, and HPV-58 were found in 80 (72.7%, 95%CI [0.63, 0.80]) women [including multiple infection], of whom nine (8.2%, 95%CI [0.04, 0.15]) women had multiple infections (Table 4).

**Table 4:** Vaccine-targeted genotype distribution based on types of infections.

Vaccine	hr HPV [n = 110]			95% CI	Single infection			Multiple infection		
	Genotype	N	%		N	%	95% CI	N	%	95% CI
Cervarix, Gardasil^+^, and Gardasil 9^+^	HPV-16	36	32.7	[0.24, 0.42]	25	22.7	[0.16, 0.32]	11	10	[0.05, 0.17]
	HPV-18	3	2.7	[0.01, 0.08]	2	1.8	[0.01, 0.07]	1	0.9	[0.00, 0.06]
Gardasil 9^+^	HPV-31	21	19.1	[0.13, 0.28]	13	11.8	[0.07, 0.19]	8	7.3	[0.03, 0.14]
	HPV-33	3	2.7	[0.01, 0.08]	2	1.8	[0.01, 0.07]	1	0.9	[0.00, 0.06]
	HPV-45	7	6.4	[0.03, 0.13]	4	3.6	[0.01, 0.09]	3	2.7	[0.01, 0.08]
	HPV-52	13	11.8	[0.07, 0.19]	6	5.4	[0.02, 0.12]	7	6.4	[0.03, 0.13]
	HPV-58	12	10.9	[0.06, 0.18]	7	6.4	[0.03, 0.13]	5	4.5	[0.02, 0.11]

+ Targets additional two low-risk HPV genotypes [HPV-6 and HPV-11].

## Discussion

As far as we know, there has been no research conducted in Ethiopia to determine the frequency of HPV genotypes that are not covered by vaccination. Our study is pioneering in this regard by investigating the prevalence of non-vaccine-targeted HPV genotypes in Ethiopia. In this study, more than half [51.8%] of the patients had genotypes not targeted by the vaccine, and 27.3% had multiple infections—a potential associated factor for cervical cancer.

Among the study participants, the prevalence of non-vaccine-targeted HPV genotypes, including multiple infections, was almost comparable to the prevalence of vaccine-targeted HPV genotypes [51.8% vs. 48.2%]. The non-vaccine-targeted prevalence was found to be comparable to the 48.6% reported in the clinical study conducted by Mónica Saccucci et al^27^. in 2018. However, it was higher than the outcomes of clinical studies carried out in the Philippines [5.1%] and France [21.6%]^26^. The findings of this research indicate that a significant number of HPV genotypes in Ethiopia are not included in the current vaccination program. Despite the implementation of the HPV vaccination program in Ethiopia for girls aged 9–14 years, targeting HPV-6, HPV-11, HPV-16, and HPV-18^28^, there are still other genotypes that are not covered.

The results of previous vaccine trials and post-implementation evaluations have shown that the HPV vaccines can offer some additional protection against HPV genotypes that are not directly targeted by the vaccines. However, the level of cross-protection varies depending on the vaccine type and HPV genotype^29,30^. In addition, the findings of cross-sectional studies done at various points in time have shown an association between multiple infections of hr HPV genotypes and cervical cancer^35,47–50^. While more research with a longitudinal study design is needed to fully understand the phenomenon, the current study found that the rate of multiple infections was 27.3% and that HPV-16, HPV-31, and HPV-35 with different genotypes had a substantial number of multiple infections. Additionally, cervical cancer is frequently linked to multiple HPV genotype infections, and non-vaccine-targeted genotypes may cause cancer to develop more rapidly and extensively when combined with vaccine-targeted genotypes [such as HPV-51 and HPV-58]^33–35^.

The HPV vaccine [Gardasil] currently being administered in Ethiopia which targets HPV-16 and HPV-18 [high-risk group] and HPV-6 and HPV-11 [low-risk group] has a chance to curtail the transmission of other non-vaccine-targeted HPV genotypes^51–53^, the cross-protection immunity provided by the vaccine will decrease over time^54^. For this reason, as stated in some studies, there is a possibility of becoming a major public health problem if preventive measures including HPV genotypes that are not the target of the vaccine are not implemented. To further support this idea, results from a 2014 clinical trial showed that non-vaccine-targeted genotypes, namely HPV35/39/51/56/59, accounted for 23–30% of CIN1, 7–14% for CIN2, 3–4% Incidence of CIN3 lesions, were the primary causes^55^. Thus, such information will greatly contribute to the selection of the type of vaccine we intend to use for cervical cancer prevention and to better cover the genotype.

Therefore, determining the prevalence of non-vaccine-targeted genotypes can serve as baseline data for future studies using a longitudinal study design. In addition, the results of this study will encourage other researchers to evaluate the role of non-vaccine-targeted genotypes in combination with other genotypes in the development of cervical cancer, as well as the effects of high numbers of non-vaccine genotypes such as HPV-35 alone and in combination with other cervical cancer-causing genotypes on cervical cancer development.

## Limitation of the study

The study was cross-sectional and did not investigate a cause-effect relationship. Nevertheless, the application of an extended genotyping method of diagnosis strengthens the findings.

## Conclusion

In the study, the prevalence of non-vaccine-targeted HPV genotypes was high, with the predominant non-vaccine-targeted genotypes of HPV-35, HPV-68, HPV-56, HPV-66, HPV-39, HPV-51, and HPV-59. More than a quarter of the study participants had multiple infections. The non-vaccine-targeted hr HPV genotypes are equally likely to be a cause of cervical lesions.

Revising the partial genotyping method currently implemented in Ethiopia with an extended or complete genotyping method would be important in determining the contemporary genotype heterogeneity level, rate of multiple infections, and the non-vaccine-targeted genotype prevalence. This will be helpful to provide evidence-based selective treatment to the patient and to evaluate the role of the non-vaccine-targeted genotype co-existence in the process of carcinogenesis.

Most importantly, in addition to the currently used qHPV vaccine, revision of the partial genotyping method will have a significant role in providing additional evidence to review and modify the current vaccination guidelines as necessary.

## Data Availability

All data supporting the results reported in the article are include in the manuscript.
